# The impact of teaching-research conflict on turnover intention: cross-level interaction effect of justice climate

**DOI:** 10.3389/fpsyg.2023.1283477

**Published:** 2023-11-29

**Authors:** Zhao Siqi, Wang Hong

**Affiliations:** ^1^School of Education, Guangzhou University, Guangzhou, China; ^2^School of Educational Sciences, Harbin Normal University, Harbin, China

**Keywords:** turnover intention, teaching-research conflict, justice climate, job burnout, career adaptability

## Abstract

**Introduction:**

Research-based on the Job Demands-Resources theory (JD-R theory) has revealed a close relationship between teaching-research conflict and job burnout. However, there needs to be more investigation into the complex relationship between teaching-research conflict and turnover intentions from the perspective of this theory. To address these gaps, this study, grounded in the JD-R theory, explores the relationships among teaching-research conflict, career adaptability, justice climate, job burnout, and turnover intention.

**Methods:**

Data collected through an online survey involving 858 Chinese university teachers, and the analysis utilized a Multilevel Structural Equation Model (MSEM) with Maximum Likelihood (ML) estimation.

**Results:**

The findings reveal that job burnout mediates the relationship between teaching-research conflict and turnover intention. Career adaptability plays a moderating role in the connection between job burnout and turnover intention. Furthermore, justice climate exhibits a cross-level interaction effect concerning the relationship between teaching-research conflict and turnover intention.

**Discussion:**

These findings offer innovative strategies for mitigating and preventing faculty turnover intention.

## 1 Introduction

As part of their performance management reforms, China’s higher education institutions have introduced a performance evaluation system based on the “publish or perish” principle. Several university leaders strictly depend on the number of academic papers as the sole criterion for evaluating faculty performance, resulting in a prevailing utilitarian trend in China’s academic environment. Educators face the persistent challenge of generating the maximum number of research papers in the shortest period. Moreover, the substantial demands of teaching and the burden of routine administrative tasks deplete the cognitive and temporal resources of many educators, aggravating the experience of the teaching–research conflict (Cao et al., 2020; Lei et al., 2020; Naz and Arshad, 2022), leading to the increasing intention to leave their positions (Kaniuka, 2020; Simms et al., 2020; Asfahani, 2022; Li and Yao, 2022).

In line with this situation, the National Teacher Development Survey conducted by Peking University’s School of Education specified that approximately 66% of higher education professionals strongly intend to leave their current roles (Du and Liu, 2019). In addition, UNESCO highlights that teacher turnover has reached unprecedented levels, and the global enthusiasm for pursuing a career in education has decreased significantly (UNESCO, 2022). This compelling evidence suggests that the above phenomenon is not unique to China but is spreading globally. More importantly, research shows that university faculty turnover diminishes academic research output and organizational appeal (Lei et al., 2020), and includes financial costs and considerable time investment (Sorensen and Ladd, 2020). This situation could undermine the effective implementation of strategies aimed at national progress in science and education (Xu, 2017). For this reason, addressing the challenge of teacher turnover has become increasingly apparent not just for higher education institutions but also for government bodies and society at large. Therefore, an in-depth study of teacher turnover in higher education is necessary.

Owing to the irreversibility of turnover behavior, scholars believe that examining turnover intention as the early psychological process of actual turnover behavior holds immense practical significance (Tett and Meyer, 1993; Bratman, 2009; Bothma et al., 2013). Turnover intention is a psychological state corresponding to an employee’s desire to leave their current position and commitment to this decision (Bratman, 2009). It has significant predictive power for assessing turnover rates (Tett and Meyer, 1993; Bratman, 2009; Bothma et al., 2013) and exhibits a strong association with multiple factors. These factors include job performance, mental health, career adaptability, burnout, organizational climate, and student academic achievement (Kaniuka, 2020; Saddam et al., 2022). Furthermore, extensive research on work–family conflict underscores the significance of role conflict as an antecedent to turnover intentions (Musadieq, 2020; Dodanwala et al., 2022). Simultaneously, scholars within the JD-R theory assert that role conflict constitutes a significant job demand (Wijayati et al., 2019; Boudrias et al., 2020). For instance, Li et al. (2019) established a robust association between teaching-research conflict, which represents a distinctive job demand, and burnout among university educators.

Nevertheless, the relationship between teaching–research conflict as a job demand and turnover intention remains underexplored. Building on the abovementioned points and considering China’s current context, this study aims to examine the complex relationship between teaching–research conflict as a specific job demand and the turnover intention of university educators, employing the JD-R theory framework. The objective is to address research gaps and recommend potential strategies for mitigating turnover intention among Chinese higher education professionals.

### 1.1 Hypothesis development

#### 1.1.1 Theoretical basis

The JD-R theory (Bakker and Demerouti, 2007; Bakker and Demerouti, 2014; Demerouti and Bakker, 2022) develops two interrelated yet distinct systems to shed light on employee behavior. Job demands include all work-related factors that deplete an individual’s physical and mental energy and are significantly correlated with adverse job performance, burnout, and diminished job satisfaction. Such as elevated work pressure, role conflict, hostile working conditions, and irregular work hours (Bakker and Bal, 2010; Xu, 2017). Hence, it is evident that teaching–research conflict, a specific form of role conflict, can classified as one of these job demands (House and Rizzo, 1972; Xu, 2017; Cao et al., 2020; Lei et al., 2020; Li et al., 2021).

Job resources encompass diverse physical, psychological, social, and organizational aspects, categorized into external and internal resources. External resources include organizational and social components like organizational climate and social support (Bakker and Demerouti, 2007; Boudrias et al., 2020). From this, justice climate, as an organizational-level phenomenon (Liao and Rupp, 2005; Rupp et al., 2007; Chaiken and Ledgerwood, 2012), represents an external resource. Internal resources, or personal resources, consist of cognitive attributes and behavioral patterns, such as self-efficacy, organizational-based self-esteem, and optimism (Xanthopoulou et al., 2007). Hobfoll (2002) acknowledged these resources as fundamental components of individual adaptability, empowering individuals to exert control over their work environment. Accordingly, career adaptability represents a personal resource that can ignite intrinsic motivation, assist in coping with physical and psychological job demands, and enhance work engagement and overall well-being. Studies confirm that job resources significantly predict extra-role performance, job engagement, and disengagement (Demerouti et al., 2001).

Indisputably, JD-R theory provides invaluable insights, clarifications, and predictive capabilities concerning workplace burnout and performance. However, it is imperative to acknowledge the current research gap, especially when considering the relationship between multiple job resources, job demands and job outcomes. Therefore, this study decisively selects career adaptability as an individual resource and justice climate as an environmental resource. It aims to enhance our comprehension of how these resources coexist and impact employee outcomes, further contributing to the enrichment of research findings within JD-R theory.

#### 1.1.2 Literature review

##### 1.1.2.1 Teaching–research conflicts and turnover intention

Teaching–research conflict, a form of role conflict arising from discrepancies in role objectives or work content misalignment between teaching and research activities (Lewin, 1935; Elton, 1986; Mooney, 2022), has been the subject of empirical investigation within the JD-R theory framework. These studies highlight that teaching–research conflict, classified as job demands (House and Rizzo, 1972; Xu, 2017; Li et al., 2019; Cao et al., 2020; Lei et al., 2020), significantly contributes to job burnout among university educators (House and Rizzo, 1972; Xu, 2017; Cao et al., 2020; Lei et al., 2020). Moreover, teaching–research conflict significantly correlated with well-being, psychological capital, job insecurity, teacher self-efficacy, and teaching motivation (House and Rizzo, 1972; Xu, 2017; Li et al., 2019; Cao et al., 2020; Lei et al., 2020; Rafsanjani et al., 2020). However, current studies considering the relationship between teaching–research conflict and turnover intention remain theoretical (Martin and Berry, 1969; Fukudome, 2014), needing extensive empirical validation. Accordingly, this study places teaching–research conflict at the forefront of its research agenda to enrich the empirical research landscape and furnish more authoritative evidence.

According to the JD-R theory, job demands often include tasks requiring considerable cognitive effort or emotionally challenging interactions with individuals (Bakker et al., 2014). These tasks expend more physical and mental energy, potentially leading to damaging consequences such as amplified stress and turnover intention (Demerouti and Bakker, 2022; Li et al., 2022). To rephrase it, teaching–research conflict, as job demands, can lead to adverse work-related outcomes, including an increased turnover intention (Daly and Dee, 2006). Notably, the context of China’s National Strategy for Promoting Science and Education emphasizes the principle that “science and technology are the primary productive forces,” leading to university faculty being increasingly coerced to prioritize scientific research. However, this sudden increase in research expectations disturbs the delicate balance between their teaching and research responsibilities, intensifying competition for time and energy (Cao et al., 2020; Estigoy et al., 2021). This intensification leads to high-intensity teaching–research conflict, triggering negative emotions such as anxiety and worry. These not only adversely affect the professional quality and self-evaluation of educators but also increase the likelihood of developing a strong turnover intention to resign (Kaniuka, 2020; Simms et al., 2020; Asfahani, 2022; Vercio et al., 2022; Lambert et al., 2023).

Considering the detrimental impact of teaching–research conflict on university educators and the existing research gaps, this study aims to examine the relationship between teaching–research conflict and turnover intention from the perspective of the JD-R theory. It also seeks to improve our understanding of the impact of teaching–research conflict and provide novel direction to alleviate educators’ role conflict and turnover intention. Therefore, we propose the following hypothesis:

*H1*: Teaching–research conflict is positively correlated with the turnover intention of university educators.

##### 1.1.2.2 Mediating role of job burnout

Job burnout is a syndrome closely linked to the work environment, characterized is typified by sustained psychological distress and comprises three core dimensions: chronic exhaustion, indicative of the depletion of an individual’s physical and emotional energy reserves; cynicism, denoting a detached and cynical attitude toward work; and diminished professional efficacy, signifying a decline in perceived competence and accomplishments within the organizational climate (Maslach et al., 2001). Li et al. (2022) and Schwab and Iwanicki (1982) revealed a substantial correlation between job burnout and role conflict. Furthermore, recent research underscores the role of job burnout as a mediating factor in the association between role conflict and turnover intention. For instance, Han et al. (2015) elucidated that role conflict effectively predicts job burnout among healthcare professionals, increasing the likelihood of their intention to leave their current employment. Similarly, Asfahani (2022) stresses the role of the exhaustion dimension of job burnout as a mediator between role conflict and turnover intention. Thus, it becomes apparent that individuals facing heightened role conflict are more likely to experience job burnout and, subsequently, their propensity toward turnover intention increases.

The JD-R theory boosts our understanding of the intricate connection between job demands (such as role conflict) and burnout. This theory states that elevated job demands are intrinsically related to health impairment, highlighting the potential risks of job burnout and subsequent health complications (Mäkikangas et al., 2020; Demerouti and Bakker, 2022; Bakker et al., 2023). Based on this theory, we can infer that in China’s science and education revitalization policy, the tension between teaching and research has become a significant stressor for educators, implying heightened job demands. Educators must balance their teaching responsibilities and research tasks to foster university growth and enrollment. However, teaching and research demand substantial mental and cognitive resources, predisposing individuals to energy depletion, deteriorating physical health, and amplified susceptibility to job burnout. This leads to some educators intending to leave to escape these experiences (Chênevert et al., 2021).

Remarkably, within the JD-R theory, prior research has frequently treated occupational burnout as a dependent variable; however, the process effects of burnout warrant further investigation. Therefore, the study considers job burnout as a mediating variable in the relationship between teaching–research conflict and turnover intention, which aim to expand upon existing research, further offering empirical support for applying the JD-R theory to clarify the connections among teaching–research conflict, turnover intention, and burnout. Accordingly, we propose the following hypothesis:

*H2*: Job burnout mediates the relationship between teaching–research conflict and turnover intention.

##### 1.1.2.3 Moderating effect of career adaptability

Career adaptability, as emphasized by scholars such as Le et al. (2019), Savickas (1997), Savickas and Savickas (2019), and Stead et al. (2021) serves as a vital psychological asset that enables individuals to navigate the complex landscape of their careers efficiently. This unique capacity empowers individuals to proactively participate in shaping their career development, establishing, and managing their professional goals, and effectively adapting to the ever-changing dynamics that often influence the course of their careers (Savickas and Savickas, 2019; Haenggli and Hirschi, 2020; Stead et al., 2021). Substantial empirical evidence consistently underlines the indispensable role of career adaptability in faculty retention and turnover (Zhu et al., 2019). Furthermore, it is significantly associated with a broad spectrum of positive career outcomes, including career success, heightened work engagement (Yang et al., 2019), refined job-related competencies (Chan, 2015), elevated self-esteem (Hamzah et al., 2021), enhanced well-being, and successful management of job-related stress (Bakker et al., 2014). Haenggli and Hirschi (2020) also observed that career adaptability, which functions as a personal resource, effectively moderates educators’ personal and occupational outcomes.

According to the JD-R theory, career adaptability is a personal resource, signifying constructive self-assessment and motivational drivers (Xanthopoulou et al., 2007; Bakker et al., 2014; Li et al., 2019). Like job resources, it is a buffer against job demands (Tremblay and Messervey, 2011; Bakker and de Vries, 2020). This theory highlights that a lack of resources complicates meeting job demands, leading to further withdrawal behavior. The long-term consequence is disengagement from work (Xanthopoulou et al., 2007; Bakker et al., 2014; Le et al., 2019). Conversely, individuals with abundant personal resources are less likely to experience job stress and burnout (Bakker et al., 2014). Additionally, Radey et al. (2023) noted that various job demands can readily lead to adverse outcomes without these personal resources. Therefore, it can be inferred that educators with a high level of career adaptability are likely to assist themselves in maintaining their professional efficacy, deriving meaning from the impact of burnout, further reducing withdrawal behavior, and thus suppressing turnover intention (Zhu et al., 2019). Conversely, educators with limited career adaptability may face challenges in efficiently managing burnout caused by job demands, potentially increasing their likelihood of intending to leave their jobs.

While existing research has examined career adaptability within the context of job demands and burnout, a research gap exists regarding the role of career adaptability in the relationship between burnout and adverse work outcomes. Thus, the present study assigns career adaptability as a pivotal variable, serving as a buffer to mitigate the influence of job burnout on turnover intention. Based on this, we propose the following hypotheses:

*H3*: Career adaptability moderates the relationship between job burnout and turnover intention.

##### 1.1.2.4 Cross-level interaction impact of justice climate

Justice climate as a unit-level phenomenon (Liao and Rupp, 2005; Rupp et al., 2007; Chaiken and Ledgerwood, 2012). Research advocates that a justice climate is a substantial job resource (Boudrias et al., 2020). More precisely, it embodies the collective perception of justice at the group level, indicative of employees’ shared perspectives on justice within the organization (Rupp et al., 2007; Li and Cropanzano, 2009; Priesemuth et al., 2013; Siswanti et al., 2020). This collective perception developed by aggregating the diverse justice-related viewpoints that employees hold (Whitman et al., 2012). Scholars rooted in social information and heuristic justice theories (Cropanzano et al., 2001; Lind et al., 2001; Chaiken and Ledgerwood, 2012) have determined the process by which a justice climate was generated. They proposed that the fundamental concept of a justice climate is comparable to the environment that individuals within an organization share. This environment comprises resources, procedures, decisions, common cognition, experiences, and organizational judgments. In simpler terms, members actively seek information about justice through social interactions and interpersonal communication in a group marked by interdependence and social interaction (Devine and Heath, 1999; Cohen, 2004; Zohar and Luria, 2004). Subsequently, they employ cognitive processes to synthesize and integrate this information, considering their own and others’ perspectives. This cognitive synthesis reinforces justice judgments and culminates in constructing a collectively shared understanding of justice (Baldwin, 1992; Meyer, 1994; Johanson, 2000; Cropanzano et al., 2001). Moreover, within the heuristic justice theory framework (Lind et al., 2001), this collectively synthesized information is used as a cognitive shortcut to evaluate fair treatment, resulting in the emergence of the justice climate as a collective phenomenon (Rupp et al., 2007; Chaiken and Ledgerwood, 2012; Whitman et al., 2012).

In recent years, there has been a widespread call for research on equality, justice, and diversity, bringing the concept of justice climate and its associated research methods to the forefront. Empirical research has systematically operationalized the notion of a justice climate as a group-level phenomenon, often involving using direct or referent shifting approaches, where employee scores are aggregated into the composite justice climate of a group (Mossholder et al., 1998; Simons and Roberson, 2003; Liao and Rupp, 2005; Priesemuth et al., 2013; Walumbwa et al., 2017; Ambrose et al., 2019), which providing this study with a robust methodological foundation to assess justice climate as a group-level variable. Moreover, justice climate research has extended its influence across various fields, including social economics, management, and public health (Whitman et al., 2012; Priesemuth et al., 2013; Rupp et al., 2017; Siswanti et al., 2020). Notably, the justice climate negative correlation with turnover intention and the experience of role conflicts (Mengstie, 2020) and acts as a moderator in the relationship between individual perceptions of justice and job burnout (Martínez-Tur et al., 2020). However, previous research has frequently disregarded justice climate’s influence on the interplay between teaching and research conflicts and its impact on turnover intention. Considering the paramount significance ascribed to educational equity by China’s Ministry of Education as fundamental to social justice, researchers are compelled to engage in comprehensive investigations of the “justice climate” within the higher education sector, further providing a solid foundation for effecting enhancements in the “justice climate” of the educational system.

According to the JD-R theory, justice climate is an external resource of job resources, enabling the achievement of work-related objectives, mitigating job demands, and diminishing the physiological and psychological tolls they impose. Based on this, justice climate, categorized as a job resource, can potentially alleviate the effects of teaching–research conflict on turnover intention. More specifically, employees are likely to cope with stress and be more willing to work when the organization fosters a justice climate. In contrast, for educators working in an environment characterized by poor justice, the lack of equal treatment further erodes their confidence in their roles within educational institutions. This disenchantment with the current working environment increases the intention to leave (Lambert et al., 2023). Thus, we propose the following hypothesis:

*H4*: The justice climate acts as a cross-level interaction in the impact mechanism of teaching–research conflict on turnover intention.

### 1.2 Current study

**Figure 1:**
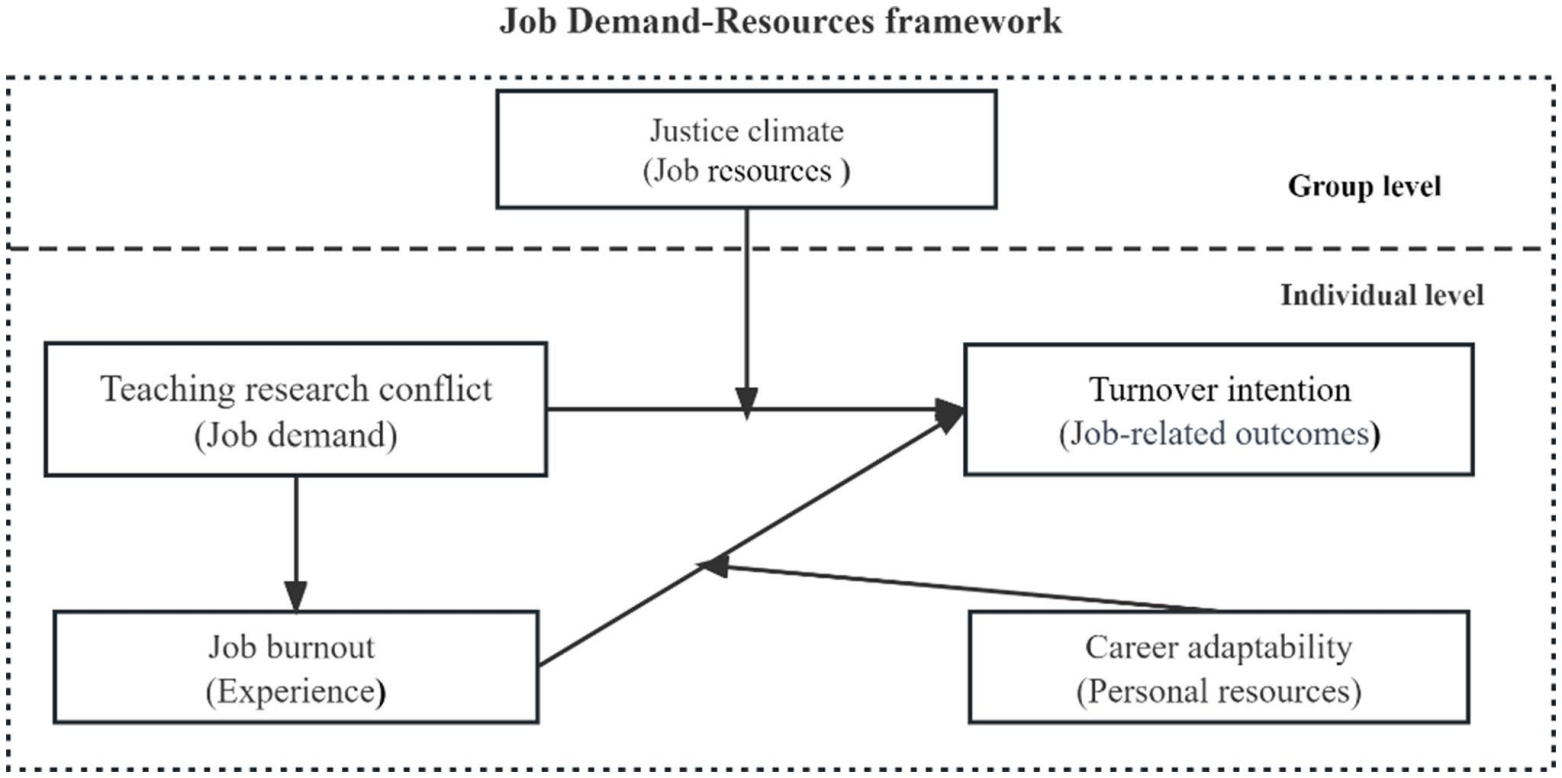
Conceptual model.

This study explores the relationships between two resources (career adaptability and justice climate), one job demand (teaching-research conflict), job burnout, and turnover intention, grounded in the JD-R theory. The conceptual model, as depicted in Figure 1.

## 2 Methods

### 2.1 Data collection and participants

This study utilized a cross-sectional design and employed convenience sampling for participant recruitment. Data was collected through an online survey administered via the Questionnaire Star network platform. Participants were initially provided comprehensive information about the study’s content and objectives, ensuring transparency. They were subsequently informed of their right to withdraw from the study at any point and for any reason. Moreover, participants completed an online consent form confirming their voluntary participation in the anonymous questionnaire. Each participant will receive a reward of 20 yuan. Ethical approval for this study was diligently sought and obtained from the author’s department.

The study involved 858 educators selected from 30 universities across diverse regions, including Heilongjiang Province, Inner Mongolia Autonomous Region, Henan Province, Guangdong Province, Shanghai, Jilin Province, Jiangsu Province, and Beijing. The number of valid responses varied, ranging from 17 to 41 per university. The participant cohort consisted of 399 male and 459 female respondents representing various academic positions, including 177 professors, 280 associate professors, and 401 lecturers. In terms of the age distribution within the overall sample (*M* = 40.66; SD = 1.43), 221 individuals were below the age of 35, 378 fell within the 36–45 age category, 197 were situated within the 46–55 age range, and 62 were aged between 56 and 65 years.

### 2.2 Instruments

#### 2.2.1 Teaching–research conflict

Teaching–research conflict was measured based on Xu’s (2017) revised Chinese Teaching–Research Conflict Scale, derived from the Teacher/Coach Role Conflict Scale (Mellor et al., 2020); the 10 items were rated on a 7-point Likert scale. The higher the score, the higher the teaching and research role conflict. Cronbach’s α was 0.74 in this study. The fit indices of the confirmatory factor analysis were as follows: *χ^2^/df* = 2.41, RMSEA = 0.03, CFI = 0.95, IFI = 0.95, TLI = 0.96.

#### 2.2.2 Turnover intention

Turnover intention was measured based on Ran et al.’s (2020) revised Turnover Intention Scale, which included six items. Using a 5-point Likert scale. The higher the score, the stronger the willingness to leave. Cronbach’s α was 0.72 in this study. The fit indices of the confirmatory factor analysis were as follows: *χ^2^/df* = 3.07, RMSEA = 0.05, CFI = 0.93, IFI = 0.92, TLI = 0.91.

#### 2.2.3 Job burnout

Job burnout was measured based on Xu et al.’s (2004) revised Job Burnout Scale, which includes 15 items. Each statement was rated on a 5-point Likert scale. Cronbach’s α was 0.88 in this study. The fit indices of the confirmatory factor analysis were as follows: *χ^2^/df* = 4.58, RMSEA = 0.06, CFI = 0.95, IFI = 0.93, TLI = 0.92.

#### 2.2.4 Career adaptability

Career adaptability was measured based on Yu et al.’s (2019) revised Career Adapt-Abilities Scale–Short Form (CAAS-SF), which included four dimensions and 12 items, and each statement was rated using a 5-point Likert scale. Cronbach’s α was 0.87 in this study. The fit indices of the confirmatory factor analysis were as follows: *χ^2^/df* = 3.62, RMSEA = 0.05, TFI = 0.98, IFI = 0.99, CFI = 0.99.

#### 2.2.5 Justice climate

Following established research on justice and teams, we employed a referent-shift consensus model to evaluate the overall justice climate (Naumann and Bennett, 2000; Cropanzano et al., 2001; Chen et al., 2007; Thornton and Rupp, 2015). In line with Priesemuth et al. (2013), justice climate was measured based on three referent-shift items from the 6-item overall Justice Scale developed by Ambrose et al. (2019) to measure the overall justice climate. Employees were asked to indicate their agreement level with statements concerning the justice of the organization’s treatment of its employees. Ratings were collected on a 5-point scale. The justice climate at the group level was subsequently aggregated based on employees’ perceptions of organizational justice. Cronbach’s α for the scale was 0.93 in this study. The fit indices of the confirmatory factor analysis were as follows: *χ^2^/df* = 2.82, RMSEA = 0.04, CFI = 0.94, IFI = 0.95, and TLI = 0.94.

### 2.3 Data analysis

SPSS software was utilized for various essential tasks, including reliability analysis, generation of descriptive statistics, execution of correlation analyses, and evaluation of common method bias analysis on the pivotal variables. Subsequently, more advanced analytical procedures were employed utilizing Mplus software, including constructing a MSEM using the ML estimator and applying the Johnson–Newman technique to perform simple slope analysis tests. Additionally, the study examined moderation and mediation effects using the error-corrected bootstrap resampling method through 5,000 iterations. This meticulous methodology facilitated the precise computation of point estimates and derivation of 95% confidence intervals to assess indirect effects. Adopting this robust and comprehensive approach, this study aimed to provide an accurate understanding of the relationships between the variables under scrutiny.

## 3 Results

### 3.1 Common method bias test

This study performed a bifactor model comparison to test for common method bias. The results showed that the five-factor model of this study had *χ^2^/df* = 5.04 and RMSEA = 0.04; the path coefficients for each factor were significant, indicating a good model fit. However, after adding the common factor G, the model fit index failed to meet measurement standards. This indicates that the model without the common factor was better than that with the added one, verifying that the study had no serious common method bias.

### 3.2 Descriptive statistics and correlation analysis

This study examined the correlation between group-level justice climate and other individual variables. The results showed that turnover intention correlated significantly with teaching–research conflict, job burnout, and career adaptability (*r* = −0.19 ~ 0.63, *p* < 0.01). Job burnout correlated significantly with teaching–research conflict (*r* = 0.48, *p* < 0.01). The justice climate group was significantly correlated with teaching–research conflict, job burnout, and career adaptability (*r* = −0.16 ~ −0.07, *p* < 0.05). Career adaptability was significantly correlated with teaching–research conflict and job burnout (*r* = −0.13 ~ −0.10, *p* < 0.05). Table 1 presents the details.

**Table 1:** Descriptive statistics and correlation analysis (*n* = 858).

	***x ± s***	1	2	3	4	5
1. Teaching-research conflict	4.04 ± 1.21	1				
2. Job burnout	3.45 ± 0.96	0.48^**^	1			
3. Career adaptability	3.01 ± 0.98	−0.10^**^	−0.13^**^	1		
4. Turnover intention	3.10 ± 0.87	0.33^**^	0.63^**^	−0.19^**^	1	
5. Justice climate group mean	3.47 ± 1.47	−0.16^**^	−0.07^*^	0.14^**^	−0.13^**^	1

^**^p < 0.01; *p < 0.05.

### 3.3 Cross-level interaction effect model test

Following Grimm’s (2017) framework, the initial step concerned assessing the feasibility of aggregating data from the individual to the group level. By the ML estimation technique within an MSEM using Mplus 8.0. The effectiveness of data aggregation was assessed using criteria such as rWG, ICC (1), and ICC (2). The results revealed that the median value of rWG for team cross-border behavior was 0.98, surpassing the threshold of 0.5, suggesting a substantial level of agreement in the aggregation process. ICC (1) was calculated at 0.015, indicating significant variance among different groups. Additionally, ICC (2) demonstrated a value of 0.84, exceeding the threshold of 0.70. These results indicated that aggregating justice climate into the group-level variable was viable. Subsequently, the study constructed multilevel path analysis models to test the research hypotheses systematically.

**Figure 2:**
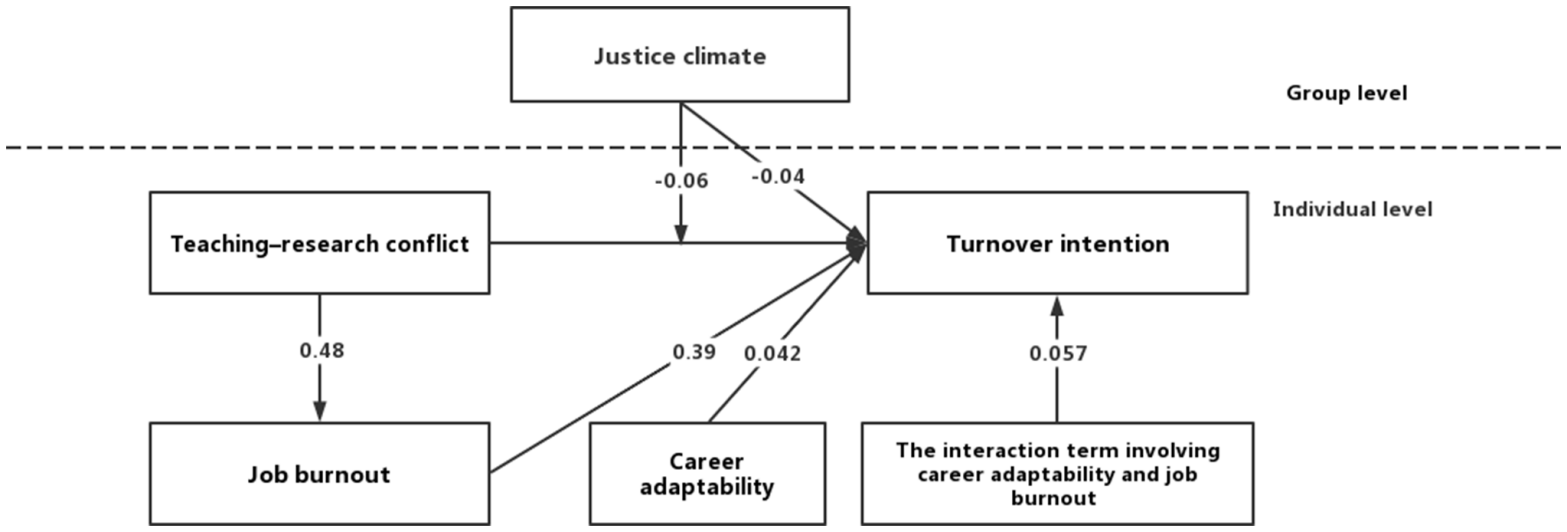
Multilevel structure equation model.

The results showed that justice climate had a significant cross-level predictive effect on the turnover intention of university educators (*β* = −0.04, *p* < 0.01). The cross-level interaction effect of justice climate on the path between teaching–research conflict and turnover intention was significant (*β* = −0.06, *p* < 0.01). The predictive effect of teaching–research conflict on job burnout was significant (*β* = 0.48, *p* < 0.01). Job burnout significantly predicted turnover intention (*β* = 0.39, *p* < 0.01). Career adaptability significantly predicted educators’ turnover intention (*β* = −0.06, *p* < 0.01). The interaction term involving career adaptability and job burnout significantly predicted educators’ turnover intention (*β* = −0.04, *p* < 0.01). The moderated mediation effect of teaching–research conflict on turnover intention was −0.02. The results demonstrated that job burnout mediates the influence of teaching–research conflict on turnover intention, and career adaptability has a moderate effect on the mediation of job burnout. Additionally, justice climate has a cross-level interaction effect on the path between teaching–research conflict and turnover intention. Details of Multilevel structure equation model is in Figure 2.

#### 3.3.1 Simple slopes of career adaptability

**Figure 3:**
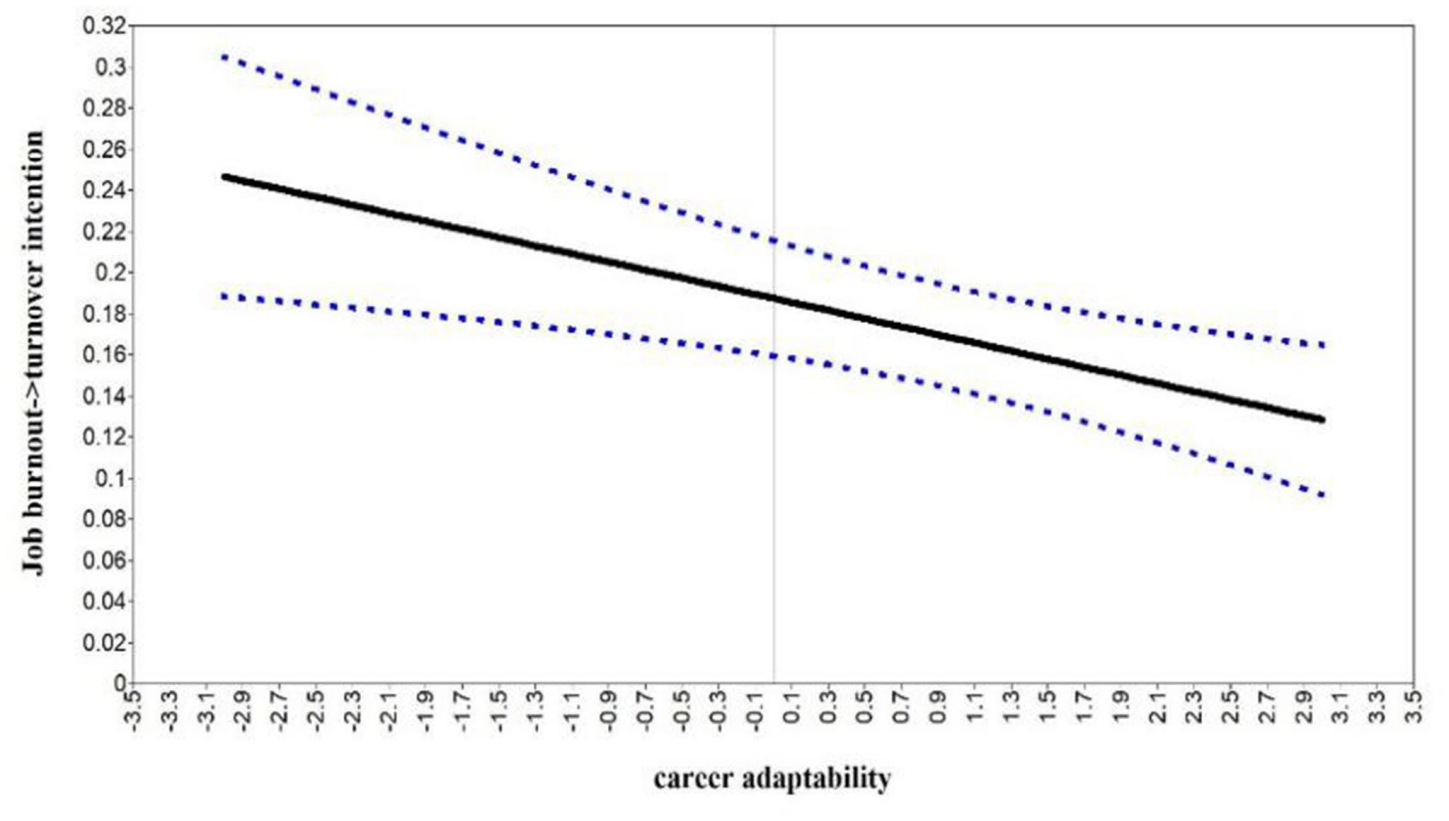
Johnson–Newman regions of significance and confidence bands for the conditional relation between job burnout on turnover intention as a function of career adaptability.

Following the recommendations of Bauer and Curran (2005), this study employed the Johnson-Newman technique to test the simple slope of career adaptability (Johnson and Fay, 1950). This technique offers a comprehensive analysis by considering the entire spectrum of career adaptability values, thus overcoming the limitation of previous studies that concentrated solely on extreme values. The result found that job burnout significantly predicted turnover intention at the entire value range of career adaptability. However, when career adaptability was low, job burnout had a more substantial effect on turnover intention (M − 1 SD; *β* = 0.43, *p* < 0.01) than on high career adaptability (M + 1 SD; *β* = 0.33, *p* < 0.01). The results indicated that the predictive effect of job burnout on turnover intention is low when the career adaptability score is high. Details of the simple slope results for career adaptability are in Figure 3.

#### 3.3.2 Simple slopes of justice climate

**Figure 4:**
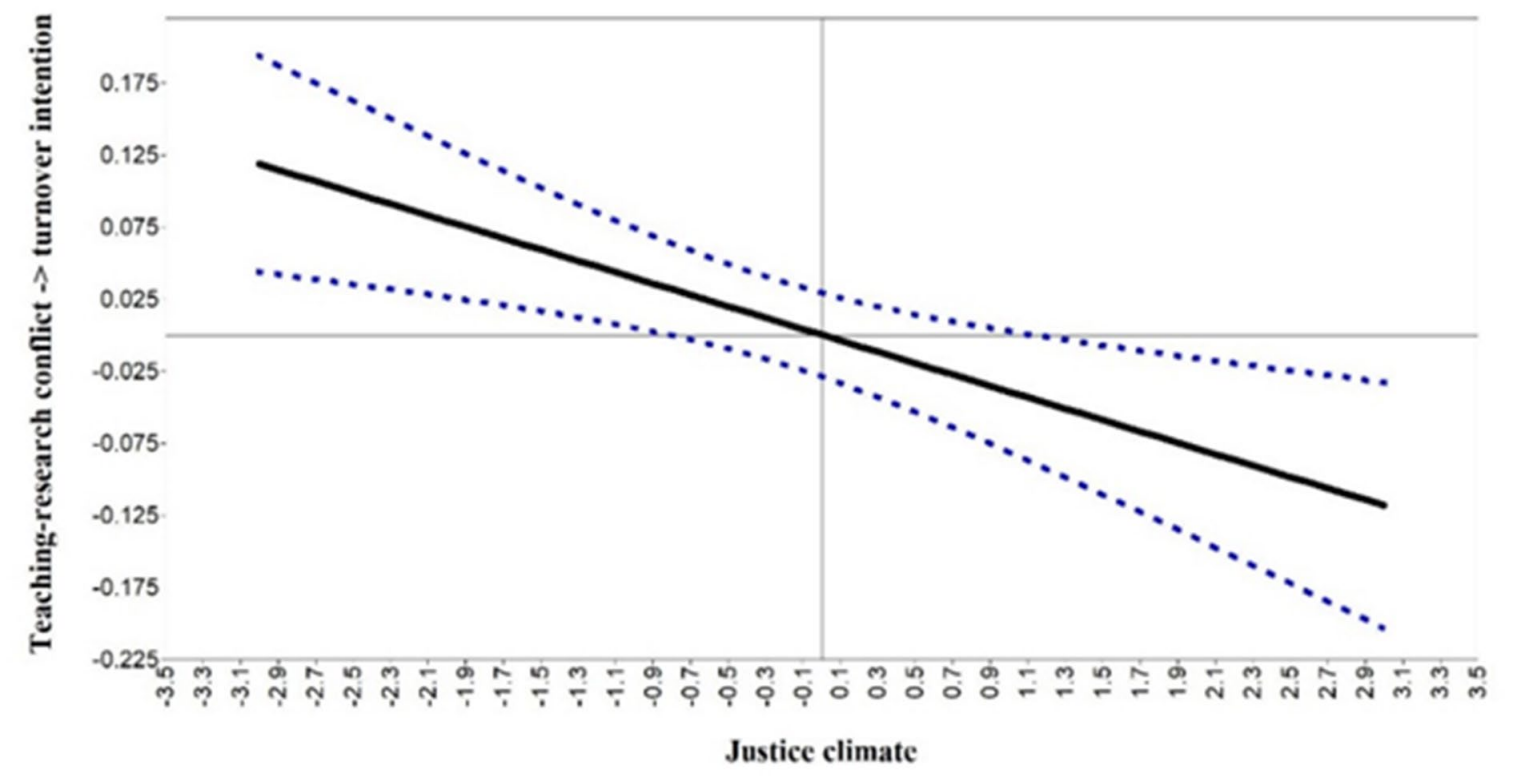
Johnson–Newman regions of significance and confidence bands for the conditional relation between teaching–research conflict on turnover intention as a function of justice climate.

The Johnson-Newman technique was employed to test the simple slope of justice climate. The results showed that the conditional effect of teaching–research conflict on turnover intention (*β* > 0.04, *p* < 0.05) was significantly positive at justice climate levels less than M-0.08 SD. When the justice climate level was above M + 1.1 SD, the teaching–research conflict had a negative path coefficient on turnover intention (*β* < −0.03, *p* < 0.05). However, when the score of justice climate was between M-0.08 SD to M + 1.1 SD, the direct effect of teaching–research conflict on turnover intention was no longer significant (−0.03 < *β* < 0.04, *p* > 0.05). Thus, the difference in the justice climate may change the direct effect of teaching–research conflicts on turnover intention. When the justice climate was less than M-0.08 SD, with the improvement of the justice climate, the positively direct effect of teaching–research conflicts on turnover intention was gradually decreased. When the justice climate was higher than M + 1.1 SD, the direct effect of teaching–research conflict on turnover intention change was negative. Furthermore, when the justice climate was M-0.08 SD to M + 1.1 SD, the direct effect of teaching–research conflict on turnover intention disappeared. Details of the simple slope results for justice climate are in Figure 4.

## 4 Discussion

### 4.1 Main effect

This study’s results highlighted that teaching–research conflict has a significant positive direct effect on turnover intention, confirming H1. This finding aligns with prior research, particularly that of Asfahani (2022), who conducted a study of 58 Saudi universities and higher education institutions. The results showed that the teaching–research conflict is positively correlated with turnover intention. These findings provide empirical support for the foundational assumption of JD-R theory, which posits that high job demands lead to adverse work-related outcomes (Mellor et al., 2020). Ongoing reform initiatives within China’s higher education sector have aggravated the inherent tensions between teaching and research roles (Xu, 2017). As underscored by Lei et al. (2020) and Wang et al. (2020), the combination of responsibilities spanning from fulfilling teaching duties to engaging in research often results in compounded workloads and resource constraints, heightening role conflicts, impeding effective task execution, fostering negative work experiences, and further intensifying educators’ intentions to leave their positions.

It is crucial to recognize that the implications of teaching–research conflict on educators’ turnover intention may extend beyond the specific Chinese higher education context. As Martin and Berry (1969) mentioned, teaching and research are inseparable facets of the roles of many university professors, transcending cultural and geographical boundaries. Educators worldwide are likely to face similar challenges, as they strive to balance their teaching and research responsibilities. Furthermore, a survey conducted by Geschwind and Broström (2014) on how three Swedish universities manage the research–teaching relationship disclosed a distinct contradiction between academic faculties’ institutional incentives and teaching requirements, underlining this issue’s universal nature. As educators’ roles continue to evolve globally, understanding the factors that contribute to their job satisfaction and retention has become a collective concern. While our study is centered on China’s unique context, the principles explained here may resonate with educators in diverse cultural settings and educational systems. Consequently, our study’s results lay the foundation for future cross-cultural research, allowing for a more profound exploration of how cultural and institutional factors influence the interplay between teaching–research conflict and turnover intention.

### 4.2 Mediation effect

This study’s results support H2, demonstrating the significant mediating role of job burnout in the relationship between teaching–research conflict and turnover intention spotlighting the vital role of job burnout as a mediating variable within the JD-R model. This finding aligns with prior research, such as Bakker et al. (2004), which demonstrated that job demands predict role performance through their impact on job burnout. However, a unique background premise sets our research apart from that of Bakker et al. (2004). Specifically, this study’s results highlight the applicability of job burnout as a mediating variable within the JD-R model in the context of Chinese higher education and establish a foundation for utilizing the JD-R theory within the Chinese academic environment.

According to this theory, job demands have consistently emerged as the primary catalyst for burnout, leading to adverse health outcomes and organizational consequences (Bakker et al., 2014). In other words, high job demands lead to a greater prevalence of maladaptive self-regulatory cognition and behavior. This progression over time may culminate in persistent burnout and harmful organizational consequences. Thus, the demanding nature of teaching and research tasks requires educators to invest significant physical and psychological effort in depleting their cognitive and time resources. This, in turn, has adverse health effects, amplifying the potential risks of job burnout and diminishing professional efficacy, as supported by Li et al. (2022) and Saddam et al (2022). Ultimately, the cumulative impact of high job demands and burnout contributes to educators’ intention to leave their positions (Sokal et al., 2020). These results lay a solid foundation for applying the JD-R theory to comprehend the psychological aspects of turnover intention within the Chinese higher education system. Therefore, education administrators should prioritize addressing teaching–research conflict and mitigating job burnout among university educators to reduce turnover intention.

### 4.3 Moderation mediating effect

This study’s results support H3, demonstrating that career adaptability moderates the relationship between job burnout and turnover intention. The results suggest that personal resources, such as career adaptability, act as a buffer to mitigate the impact of high-level job burnout on adverse job outcomes rather than merely moderating the experience of job burnout resulting from teaching–research conflict. This finding aligns with that of Rudolph et al. (2021), who found that occupational adaptability moderates the negative impact of radical occupational changes on perceived adaptive effectiveness. These results align with the JD-R theory, which postulates that employees with ample resources are likely to have positive work experiences, greater self-evaluations, enhanced performance, improved job satisfaction, and reduced burnout (Bakker et al., 2014; Savickas, 2023). Consequently, they are less likely to consider turnover (Zhu et al., 2019). In simpler terms, when educators experience job burnout due to managing teaching–research conflict, those with high career adaptability proactively seek new opportunities, explore diverse research methods, and continuously augment their skills, injecting renewed vigor into their career trajectory (Xanthopoulou et al., 2007; Bakker et al., 2014; Le et al., 2019; Rasheed et al., 2020). However, educators with low career adaptability struggle to manage their burnout experience caused by job demands efficiently, leading to higher turnover intention.

Therefore, in China’s rapidly evolving economic and social landscape, this study underscores career adaptability’s pivotal role in mitigating the effects of teaching–research conflict and job burnout on turnover intention. As individuals with personal agency, university educators should strive to harness their resources to assuage burnout, further inhibit turnover intention, safeguard their physical and mental well-being, and boost their professional development within their work context.

### 4.4 Cross-level interaction effect

This study’s results reveal a significant cross-level interaction effect of justice climate within the context of the relationship between teaching–research conflict and turnover intention, thus supporting H4, aligns with Tangirala and Ramanujam (2008), who verified the cross-level implications of procedural justice climate on employee silence, thereby confirming the pivotal role of justice climate as a job resource influencing individuals at the group level. Notably, the simple slope analysis results revealed a distinct pattern. When the justice climate falls below M-0.08 SD, teaching–research conflict significantly and positively drives turnover intention. Conversely, when the justice climate exceeds M + 1.1 SD, it negatively impacts turnover intention. Within the justice climate range of M-0.08 SD to M+ 1.1 SD, the influence of teaching–research conflict on turnover intention becomes statistically insignificant. It indicates that as the justice climate improves, there is a dynamic shift in the relationship between teaching–research conflict and turnover intention, moving from positive to negligible and ultimately to negative. It introduces a novel and noteworthy possibility that, as the justice climate improves, teaching–research conflict no longer leads to an intention to leave; instead, it deters educators’ turnover intention. This may hint at the curious effect of the conflict between judicial climate and pedagogical research, implying that the role of pedagogical research conflict has changed. Based on the JD-R theory, we infer that a high-level justice climate helps educators transform teaching–research conflict into a driving force for self-development, incentivizing educators to remain in academic institutions and diminishing their intentions to leave.

The results validate a fundamental premise of the JD-R theory in the Chinese context, which underlines the critical role of a justice climate as a job resource in mitigating the adverse effects of job demands on individual stress levels, promoting professional development, and facilitating goal achievement. Furthermore, the shift in the direction of the influence of teaching–research conflict and turnover intention is significant because it indicates whether teaching–research conflict serves as a catalyst for educators to leave or whether an external motivator for self-improvement depends on the justice climate within their institutions. Specifically, a high justice environment assuages the adverse impact of job demands and offers a safety net for educators in coping with teaching–research conflict. Additionally, it can transform teaching–research conflict into a driving force for self-development, incentivizing educators to remain in academic institutions and weakening their turnover intention (Lambert et al., 2023). In contrast, university educators with a low level of fairness are likelier to leave their work environment faster due to the impact of teaching–research conflict. This finding implies that an unfair working climate can lead to unequal negative emotions among educators, resulting in dissatisfaction with their current work environment and a higher likelihood of resigning (Baillie, 2009).

Furthermore, the results indicate that when individual and environmental resources coexist, they assume distinct roles in shaping employee turnover intention through varying mechanisms. For instance, in this study, adaptability moderates the relationship between burnout and turnover intention. It is evident that when individual and environmental resources coexist, the former, including occupational adaptability, tends to address job demand-induced burnout, thereby enhancing employees’ work. In contrast, environmental resources can directly counteract the adverse consequences of job demands. These results enrich our understanding of existing theories and indicate that personal and environmental resources may uniquely mitigate the negative impacts of job demands.

In summary, this study’s comprehensive analysis of the cross-level moderating effect of the justice climate emphasizes the transformative influence of an improved justice climate on the relationship between teaching–research conflict and turnover intention. This outcome underscores the significance of the justice climate within Chinese educational institutions, as it can determine whether teaching–research conflict acts as a catalyst for educators to leave universities or as an external motivator for self-improvement. Additionally, it advances current research, boosting our understanding of the role of work-related resources, such as the justice climate, in mitigating the impact of job demands while furnishing valuable insights for future investigations into the complex relationship between these factors.

### 4.5 Implications

This study has several noteworthy implications. First, rooted in the JD-R theory, this study has meticulously revealed the intricate mechanisms underpinning the relationship between teaching–research conflict and turnover intention among Chinese university faculty. This validates the applicability of the JD-R theory within the Chinese academic context. Moreover, this study introduced the significant role of job burnout as a mediating factor in this relationship. It highlights the potential for organizations to reduce educators’ turnover intention by addressing teaching–research conflict and implementing targeted interventions to mitigate job burnout. Additionally, our research underlines the moderating impact of career adaptability, offering practical guidance for organizations to develop measures that reduce educator burnout and turnover intention by enhancing career adaptability. The study emphasizes the significance of the justice climate within educational institutions and determines whether teaching–research conflict catalyzes educators to leave universities or as an external motivator for self-improvement. It advances existing research, enhancing our understanding of the role of work-related resources, particularly the justice climate and job demands. These results further reinforce the evidence for the urgency of fostering equitable environments in higher education. Finally, the study’s findings disclose that when individual and environmental resources coexist, they assume distinct roles in shaping employee turnover intention through varying mechanisms. This diversity in resource roles provides innovative ideas for formulating intervention methods to reduce turnover intention based on resource factors.

In terms of practical application, organizations can utilize these findings to help reduce turnover intention among employees, especially within the academic sector, where educator retention is crucial. It is essential to recognize the mediating function of job burnout and the need for relevant departments to provide professional psychological counseling services for educators. The focus should be on mitigating burnout and lowering turnover intention by providing such services. Furthermore, considering the pivotal role of career adaptability in the professional setting, educators and academic institutions should prioritize the enhancement of career adaptability. By effectively harnessing this resource, the adverse effects of burnout stemming from the conflict between teaching and research on educators can be mitigated. Finally, recognizing the vital importance of promoting fairness and equity within colleges and universities, concerted efforts should be made to promote equitable practices. It involves implementing measures and policies that ensure fairness, equal treatment, and impartial practices in educational institutions, ultimately contributing to faculty members’ well-being, thus inhibiting turnover intention.

### 4.6 Limitations

This study has limitations warranting acknowledgment to provide a clear understanding of the research’s scope and avenues for future investigation. First, the cross-sectional design employed in data collection limits the capacity to establish causal relationships. While associations between variables have been identified, further research should use longitudinal or experimental methods to examine the dynamic interplay among variables. Second, this study’s findings are rooted in the context of Chinese universities, with distinct cultural norms, academic traditions, and institutional practices. This context-specific focus raises questions about the findings’ generalizability to diverse cultural and regional contexts. Future research should encompass a broader array of cultural and regional contexts. Finally, despite this study’s careful consideration of numerous variables, the influence of unmeasured variables remains a possibility. Other factors within and outside the workplace could influence the relationships under investigation. Future research should, therefore, explore these unmeasured variables to refine the model.

## Data availability statement

The original contributions presented in the study are included in the article/supplementary material, further inquiries can be directed to the corresponding author.

## Ethics statement

The studies involving human participants were reviewed and approved by the Human Subjects Review Committee at Guangzhou University. The studies were conducted in accordance with the local legislation and institutional requirements. Written informed consent for participation in this study was provided by the participants’ legal guardians/next of kin. Written informed consent was obtained from the individual(s) for the publication of any potentially identifiable images or data included in this article.

## Author contributions

ZS: Writing – original draft, Writing – review & editing. WH: Methodology, Writing – review & editing.
